# Medicine as a Mission: Ida Sophia Scudder's Contributions to Indian Healthcare

**DOI:** 10.7759/cureus.75064

**Published:** 2024-12-03

**Authors:** Helan Rajan, Nidhi Shree, S Johnson, Prerna Verma, Deepu Palal

**Affiliations:** 1 Otolaryngology-Head and Neck Surgery, Dr. D. Y. Patil Medical College, Hospital and Research Centre, Dr. D. Y. Patil Vidyapeeth, Pune, IND; 2 Community Medicine, Dr. D. Y. Patil Medical College, Hospital and Research Centre, Dr. D. Y. Patil Vidyapeeth, Pune, IND

**Keywords:** christian medical college vellore, ida sophia scudder, indian missionary hospital, medical education, medical service, public health and social work, women's healthcare, women's medical education, historical vignette

## Abstract

This review article examines the life and medical contributions of Dr. Ida Sophia Scudder (1870-1960), a pioneering American physician and missionary who significantly impacted healthcare in India. Born into a family of medical missionaries, Scudder initially resisted following in her family's footsteps. However, a transformative experience during a visit to India led her to pursue a medical career and dedicate her life to improving women's health in the country. The article traces Scudder's journey from her medical education at Cornell University to her return to India, where she established the first women's medical college in the country-Christian Medical College (CMC) in Vellore. We analyze her innovative approaches to healthcare delivery, including developing the "roadside clinics" that brought medical care to rural areas and her efforts to train Indian women as doctors and nurses. Scudder's contributions extended beyond direct patient care. Her work in medical education, public health initiatives, and advocacy for women's rights in healthcare had far-reaching effects on the Indian medical landscape. The review also discusses the lasting impact of her efforts, including the continued influence of the institutions she founded and the generations of healthcare professionals she inspired. By examining Scudder's life and work in early 20th-century India, this article highlights the intersection of medicine, mission work, and social reform. It argues that Scudder's legacy lies not only in the lives she directly touched but also in her role in shaping modern healthcare practices and medical education in India.

## Introduction and background

**Figure 1:**
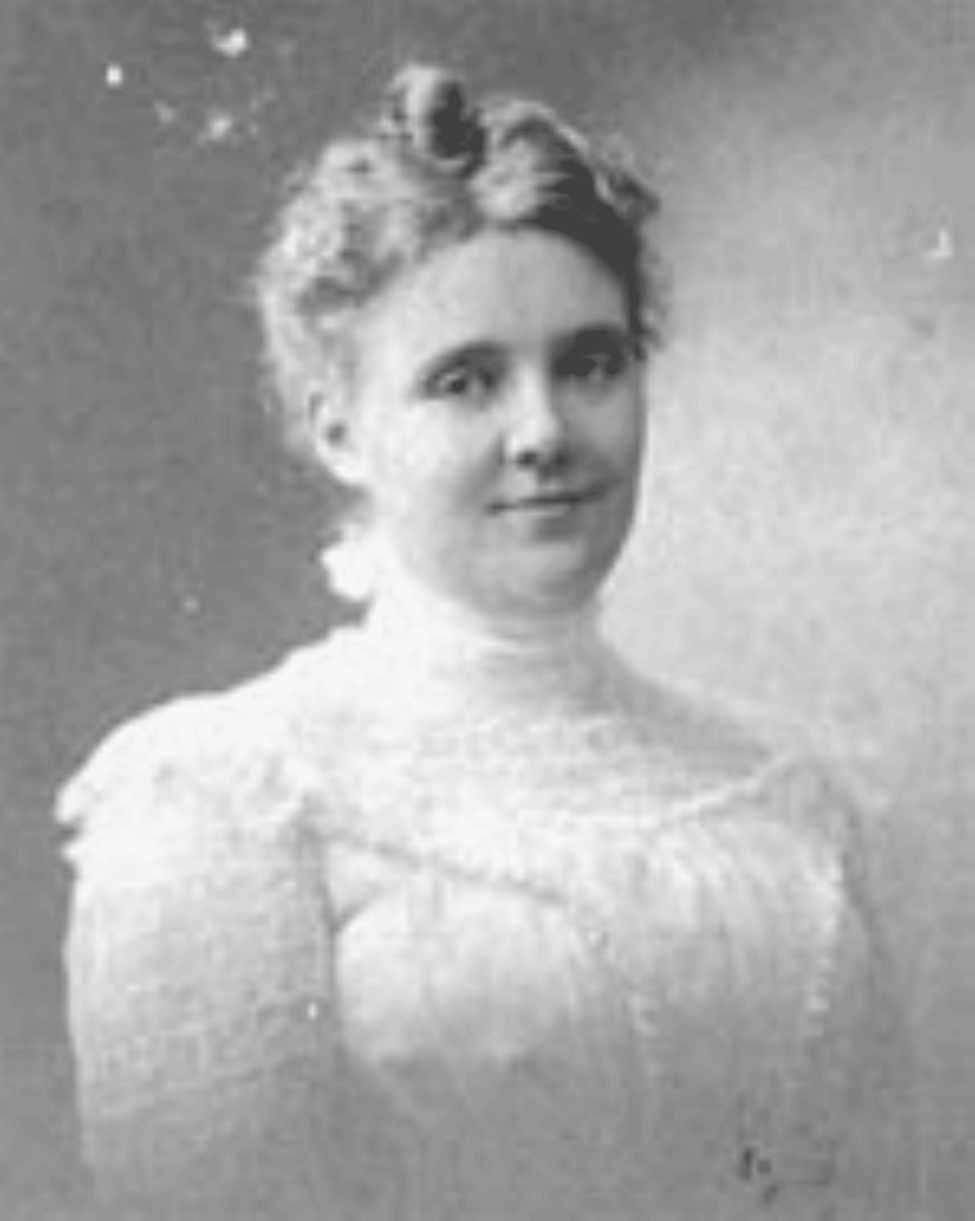
Dr. Ida Sophia Scudder Source: Wikipedia, The Free Encyclopedia (public domain) [2]

Dr. Ida Sophia Scudder (December 9, 1870-May 24, 1960) (Figure 1), an extraordinary physician, was a third-generation American medical missionary who dedicated her life to women’s rights and public health in India during the late 19th and early 20th century [1]. Her life’s work left an indelible mark on healthcare, medical education, and public health. Born into a family of American missionaries, Dr. Scudder initially resisted the medical path. However, a transformative experience in her youth ultimately redirected her life's course, positioning her as a pioneer in women’s healthcare. She was also the founder of Christian Medical College (CMC) in Vellore, India, a world-renowned institution that continues her legacy of service and compassion today [1].

## Review

Early life and call to medicine

Born in 1870 in Ranipet, India, Ida was the daughter of John Scudder, a medical missionary. The Scudder family had a long history of missionary work in India, spanning several generations. However, Ida initially had no desire to follow in her family’s footsteps. She was eager to return to the United States and live detached from the hardships she witnessed growing up in India [3]. The turning point in her life came during a visit to her family in India in 1890. One fine night in 1894, she received her call, the famous “three knocks in the night,” where three men came to her seeking medical help for their wives who were in labor. All three women died due to a lack of medical care, as local customs forbade male doctors from attending to women. This deeply moved Ida; she saw it as a divine calling to become a physician and dedicate her life to improving healthcare for women in India [4].

Education and medical training

Determined to make a difference, Ida returned to the United States to pursue medical studies. She enrolled in the Women’s Medical College of Pennsylvania and later transferred to Cornell Medical College, where she graduated in 1899 in the first class. She was one of the few women in her class who defied societal expectations of the time. Upon completing her medical training, Dr. Scudder returned to India, equipped with the skills and knowledge to serve the people who had inspired her calling [5].

Founding of CMC, Vellore

Upon returning to India, Dr. Scudder initially set up her work area in Vellore. For two years, she treated female patients in her father’s bungalow in Vellore, South India. She realized the dire need for a structured medical facility and expanded her vision. In 1900, she founded the Mary Taber Schell Memorial Hospital, named after a benefactor who supported her mission. The opening of the 40-bed Mary Taber Schell Memorial Hospital was the beginning of the realization of Ida’s vision-that women should have the same access to quality and compassionate healthcare as men, regardless of religion or the ability to pay for it. Dr. Scudder performed her first operation with an assistant who was her butler's wife, and within some time, she became a noted surgeon. By 1906, the number of patients she treated annually rose to 40,000. Her work garnered attention and financial support, allowing her to expand her efforts [6] further.

In 1918, Dr. Scudder established a medical school with a vision for women doctors, which finally evolved into CMC, Vellore. The college was groundbreaking as it trained women to become doctors at a time when societal norms did not encourage women's education, let alone their participation in medicine. The college later opened its doors to men, becoming a co-educational institution [6,7].

Impact on Indian medical education and public health

Dr. Scudder’s contributions to medicine extended far beyond CMC, Vellore. She introduced revolutionary changes in Indian medical education by advocating for the inclusion of women in the medical profession. She also emphasized the importance of preventive care and public health measures, which were critical in reducing maternal and infant mortality in the region. Her work in rural healthcare, where she focused on the underserved, is considered one of her most significant contributions [8]. Through CMC, she established the first-ever medical missionary training program in India, which has produced countless healthcare professionals who continue to serve in rural and underserved communities across the country. Her emphasis on compassionate care and her innovative approach to medical education have made CMC one of the leading medical institutions in the world [5,8].

Legacy and lasting influence

Dr. Ida Scudder passed away on October 24, 1960, but her legacy endures through CMC, Vellore, which remains a beacon of hope and healing for patients from all walks of life. The institution continues to uphold the values of compassion, service, and excellence in medical education that Dr. Scudder championed. Her contributions to Indian medical education and healthcare have had a lasting impact, particularly in empowering women to pursue medical careers [7]. To this day, CMC remains a leading medical institution in India and is renowned for its medical research, education, and healthcare services. Dr. Scudder’s dedication to serving humanity through medicine, mainly her focus on providing care to women and the underserved, set a precedent for healthcare workers worldwide [9,10].

Dr. Scudder's emphasis on women's empowerment in healthcare has had a lasting impact, inspiring generations of women to pursue medical careers. Her efforts have paved the way for the increased representation of women in the medical profession, which continues to grow even today. Her maternal and child health initiatives have also significantly improved health outcomes across the region [10]. Moreover, Dr. Scudder's legacy extends beyond India. Her pioneering work has inspired healthcare workers and educators worldwide to prioritize compassionate care and address the needs of marginalized communities. The principles she championed, integrity, empathy, and service, continue to guide healthcare practices globally [3].

## Conclusions

Dr. Ida Scudder’s life was a testament to the transformative power of compassion and service. Her unwavering commitment to improving healthcare, particularly for women and underserved populations, reshaped the medical landscape in India. She was the epitome of service, sacrifice, and compassion. She transformed the medical landscape of India, particularly for women, and left behind a powerful legacy of healing and education. Her life shows the difference one can make when driven by compassion and purpose. Through founding CMC, she established an enduring institution that trains healthcare professionals and serves as a model for compassionate, community-focused care. Today, CMC, Vellore, is a tribute to Dr. Scudder’s vision and dedication. Her legacy serves as an inspiration for future generations of healthcare professionals, reminding us of the profound impact one individual can have when driven by purpose and a desire to serve humanity.

## References

[1] Vellore Christian Medical College Foundation: Aunt Ida, Dr. Ida Sophia Scudder Founder of CMC Vellore (2024). Vellore Christian Medical College Foundation: Aunt Ida, Dr. Ida Sophia Scudder founder of CMC Vellore. https://www.cmch-vellore.edu/aunt-ida/.

[2] (2024). Ida S. Scudder. https://en.wikipedia.org/wiki/Ida_S._Scudder.

[3] Wilson Wilson, Clarke D (1994). Mission legacies: biographical studies of leaders of the modern missionary movement. Missiology.

[4] Azariah I (1990). Dr. Ida Scudder and the revolution in medical education. JSTOR.

[5] Scudder IS (1951). Scudder of Vellore: The Life Story of Ida Sophia Scudder. https://archive.org/details/idasscudderofvel0000jeff/page/n319/mode/2up.

[6] Mary TJ (2019). A historical study on the contribution of Dr. Ida Scudder-1870-1960. Think Ind J.

[7] George RM (2014). Calling, conflict and consecration: the testament of Ida Scudder of Vellore. Ch J Glo Hea.

[8] Wilson DC (1976). Dr. Ida: passing on the torch of life. USA. Friendship Press for the Vellore Christian Medical College.

[9] Paterson R (2024). An extraordinary physician: Ida Scudder. Field Partner International.

[10] Dr. Ida S (2024). Dr. Ida S. Scudder Oration Christian Medical College, Vellore. https://www.presidentofindia.gov.in/dr-apj-abdul-kalam/speeches/dr-ida-s-scudder-oration-christian-medical-college-vellore.

